# Optimization of Management Zone Delineation for Precision Crop Management in an Intensive Farming System

**DOI:** 10.3390/plants11192611

**Published:** 2022-10-04

**Authors:** Yifan Yuan, Bo Shi, Russell Yost, Xiaojun Liu, Yongchao Tian, Yan Zhu, Weixing Cao, Qiang Cao

**Affiliations:** 1 National Engineering and Technology Center for Information Agriculture, MOE Engineering and Research Center for Smart Agriculture, MARA Key Laboratory for Crop System Analysis and Decision Making, Jiangsu Key Laboratory for Information Agriculture, Collaborative Innovation Centre for Modern Crop Production Co-Sponsored by Province and Ministry, Nanjing Agricultural University, Nanjing 210095, China; 2 Department of Tropical Plant and Soil Science, University of Hawai’i at Manoa, Honolulu, HI 96822, USA

**Keywords:** soil variable, MULTISPATI-PCA, Gaussian mixture model, management zone, clustering model composites

## Abstract

Soil is characterized by high spatiotemporal variability due to the combined influence of internal and external factors. The most efficient approach for addressing spatial variability is the use of management zones (MZs). Common approaches for delineating MZs include K-means and fuzzy C-means cluster analysis algorithms. However, these clustering methods have been used to delineate MZs independent of the spatial dependence of soil variables. Thus, the accuracy of the clustering results has been limited. In this study, six soil variables (soil pH, total nitrogen, organic matter, available phosphorus, available potassium, and soil apparent electrical conductivity) were used to characterize the spatial variability within a representative village in Suining County, Jiangsu Province, China. Two variable reduction techniques (PCA, multivariate spatial analysis based on Moran’s index; MULTISPATI-PCA) and three different clustering algorithms (fuzzy C-means clustering, iterative self-organizing data analysis techniques algorithm, and Gaussian mixture model; GMM) were used to optimize the MZ delineation. Different clustering model composites were evaluated using yield data collected after the wheat harvest in 2020. The results indicated that the variable reduction technologies in conjunction with clustering algorithms provided better performance in MZ delineation, with average silhouette coefficient (ASC) and variance reduction (VR) of 0.48–0.57, and 13.35–23.13%, respectively. Moreover, the MULTISPATI-PCA approach was more conducive to identifying variables requiring MZ delineation than traditional PCA methods. Combining MULTISPATI-PCA and the GMM algorithm yielded the greatest VR and ASC values in this study. These results can guide the optimization of MZ delineation in intensive agricultural systems, thus enabling more precise nutrient management.

## 1. Introduction

Precision agriculture is important in enhancing crop productivity and increasing nutrient use efficiency [1]. Numerous studies have applied site-specific nutrient management to large-scale farmland [2,3]. However, few investigations have been conducted on medium- and small-scale farms, especially in densely populated countries such as China and India. In these countries, small- and medium-sized farms account for 72–80% of the arable land [4]. In addition, various management measures by farmers typically result in high spatial variability in soil nutrients. Moreover, the characteristic village farming systems in China, commonly 0.3–0.5 ha per farm, greatly increases the need for precision management and especially so when managed together for large-scale production. Therefore, precision agriculture for use in small, extremely diverse fields of farms in China needs to be further explored. Understanding soil spatial variability is a prerequisite for precision agriculture [5], and management zone (MZ) delineation has been widely used to address the high spatial variability of soil variables [2,6,7]. 

Management zones (MZs) have been delineated based on a variety of data: soil chemical properties [7,8], soil physical properties [9,10], apparent soil electrical conductivity (ECa) [11], historical yield data [12], satellite imagery [4], and others [13]. For medium- and small-scale farms, soil sampling is key to soil nutrient management [14]. With the development of sensor technology, proximal sensing has shown potential for delineating MZs for medium- and small-scale farms. Although researchers have demonstrated that individual factors have the potential to usefully and efficiently delineate MZs, no single soil or crop factor can completely and sufficiently characterize the complexity of crop growth and productivity [15]. Therefore, in this study, measured soil values and ECa from a proximal sensor were used to delineate MZs. Measured yield data were used to evaluate the effectiveness of the delineation.

The delineation of MZs involves many variables and observations, which potentially lead to challenges in computation, data management, and field operations. Consequently, many studies have focused on variable reduction methods to define MZs [16,17,18]. Some researchers have used principal component analysis (PCA) to achieve a suitable reduction in data dimensionality for MZ delineation [19,20,21]. However, the traditional PCA approach ignores the spatial pattern of the measured values when carrying out the classification, and may not be able to obtain centralized MZs in space. Gavioli et al. evaluated the efficiency of spatial correlation analysis (SC), PCA, and multivariate spatial analysis based on Moran’s index PCA (MULTISPATI-PCA) when used jointly with the fuzzy c-means algorithm to define MZs [16]. Based on the abovementioned methods, these scientists proposed a new method (MPCA-SC) and concluded that the approach effectively reduced the number of variables without losing important information. Their studies were designed to determine the optimal variable reduction method. However, the influence of variable reduction and weight assignment on MZ delineation needs to be further explored.

Common clustering algorithms can be classified into two categories. The first clustering algorithm uses distance as the evaluation index of similarity to divide MZs, such as K-means [22,23], iterative self-organizing data analysis technique algorithm (ISODATA) [11,21,22], and fuzzy c-means clustering algorithm (FCM) [16,17,24]. FCM delineated MZs well in most cases, as confirmed in the aforementioned studies. A well-known approach is the extension of K-means, named ISODATA [25]. The ISODATA approach is a sample and quick clustering approach that does not require the artificial selection of the number of clusters. Another category of clustering algorithms is based on models [26]. The Gaussian mixture model (GMM) is the most commonly used classification method and is based on the data distributions used in machine learning [27]. In addition, the GMM is widely used in the industrial field and has achieved good clustering results. However, the GMM approach is significantly influenced by the size of the dataset and the correlation among the variables [22]. Therefore, further research is needed to test the MZ delineation performance of GMM in study areas of different scales and datasets of different sizes. The MZ delineation performance of the GMM approach in intensive farming systems clearly merits further research. In addition, clustering algorithms have been used independently to delineate MZs in previous studies. Most clustering algorithms have ignored the spatial structure of soil variables, and unimportant variables may influence MZ delineation, thereby limiting the accuracy of delineating MZs.

Therefore, this study is based on the hypothesis that the GMM algorithm has great potential for MZ delineation in intensive farming systems, and that model composites, that is, the variable reduction technologies in conjunction with clustering models, might not only achieve data dimensionality reduction, but also optimize MZ delineation. Thus, the objectives of this study were to (1) analyze the spatial variation characteristics and quantify the spatial dependence of soil variables; (2) explore the influence of variable reduction and weights assignment on MZ delineation; (3) assess the potential of the GMM algorithm for MZ delineation in an intensive farming system; and (4) evaluate the MZ delineation performance of a variety of clustering model composites and select the optimal one using several quality assessment indices.

## 2. Results

### 2.1. Variability of Soil Properties

The classical statistics for all the soil variables and measurements are presented in Table 1. The soil in the study was extremely variable in terms of AP and AK, with AP values ranging from 5–261 mg kg^−1^ and AK values ranging from 89–323 mg kg^−1^. The concentrations of TN and OM were medium and much less variable. Wilding (1985) ranked soil properties with a coefficient of variation (CV) lower than 15% as in the low variability class, while values between 15% and 35% were moderately variable [28]. When the CV was above 35%, the variability was strong. Based on these criteria, the heterogeneity of pH was ranked weak, with a CV of 3.5%. TN, OM, AK, and yield were classified as moderately variable, with CVs ranging from 16.16–33.33%. Available soil P was ranked highly variable, with a CV of 91.43%. Available soil P was also characterized as having the widest range of values among the variables.

Analysis of the data distribution of each variable revealed that TN, OM, and AK followed a normal distribution, with the skewness and kurtosis close to 0 and 3, respectively. However, AP and ECa data were characterized by a right-skewed distribution and pH data were characterized by a left-skewed distribution. In addition, AP, ECa, and pH data were thus transformed to approximate a normal distribution by logarithmic transformation. The original data for pH, TN, OM, AK, and logarithmically transformed AP and ECa data were then used for geostatistical analysis. A global Moran’s index (Moran’s I) was used to describe the degree of spatial autocorrelation of soil variables. Soil variables and yield data revealed a positive spatial autocorrelation, with global Moran’s I ranging from 0.11–0.52.

### 2.2. Analysis of the Spatial Dependence of Soil Properties

**Figure 1:**
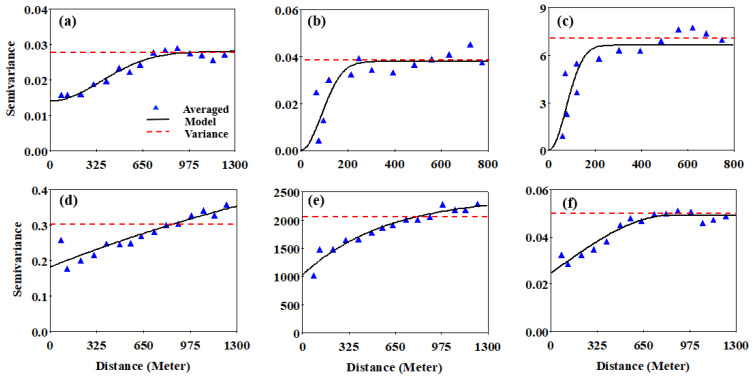
Variograms and respective models of the measured soil variables and their respective variograms models. (**a**) Soil pH, Gaussian, (**b**) TN, Gaussian, (**c**) OM, Gaussian, (**d**) AP, exponential, (**e**) AK, exponential, (**f**) ECa, spherical. The sample variance of the entire area is represented by the dotted line in each figure. Parameter estimates of the respective models are given in Table 2. Caution: note the differing scaling of the x-axis and y-axis of each figure.

The results of the semivariogram analysis are presented in Table 2 and Figure 1. The best-fitting models to characterize the change in semivariance with the distance between soil samples. The soil properties differed sharply according to this characteristic of spatial dependence. The Gaussian model most accurately characterized the semivariance and variability of soil pH, TN, and OM. An exponential model best characterized the semivariograms of AP, AK, and yield, whereas the best-fitting model for soil ECa was a spherical model. Selecting the best-fitting model of the semivariogram is important because it provides estimates of the nugget variance. Nugget variance values characterize and reveal the micro-variability of the random variables. In this study, the nugget variance values were low for soil pH, TN, OM, AP, and ECa (ranging from 0.00–0.18) and high for AK and yield (varying from 1000–1029), which, when compared with the associated values of the “sill”, characterize the spatial dependence. As indicated in Table 2, the geostatistical analysis indicated that all soil variables, as well as crop yield, were at least moderate to strong in spatial dependence, indicating the importance of soil sample spatial location. Soil TN, OM, and yield were characterized by strong spatial dependence, whereas AP was classified as moderate to strong in spatial dependence. Soil pH, AK and ECa were characterized by moderate spatial dependence. The values of soil variables ranged from 180 (for OM) to 1770 (for AK). Beyond this value, the semivariance of a specific soil property approximates that of the entire sample area, indicating no relationship between the values at that distance or greater. These values provide a guide to the required sample grid size to best characterize spatial dependence for more efficient future sampling [29].

**Figure 2:**
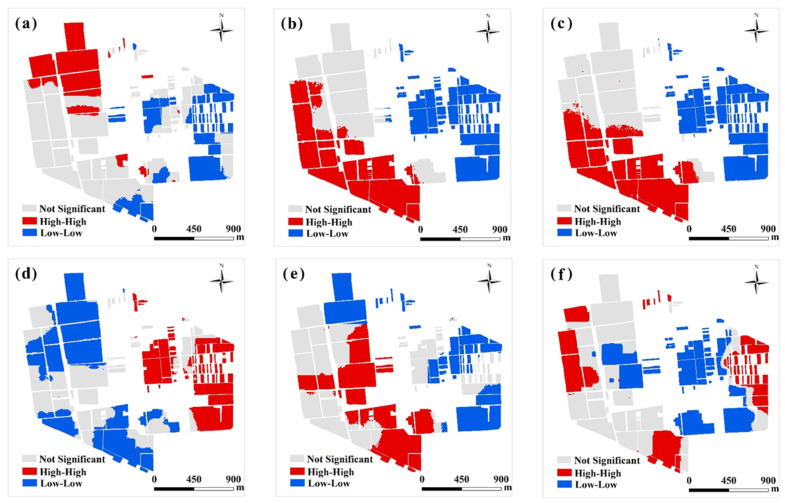
Spatial pattern maps for six different variables by visualizing the local indicators of spatial association (LISA). (**a**): pH; (**b**):TN; (**c**):OM; (**d**): AP; (**e**): AK; (**f**): ECa. Not significant: the statistic is not significant at 0.05 level, which were not referenced; High–High: high values in a high value neighborhood; Low–Low: low values in a low value neighborhood.

The global Moran’s I provides a global estimate of spatial autocorrelation among all variables, as shown in Table 1. In contrast, the local Moran’s I identifies the specific locations, enabling the spatial clustering pattern to be examined. The local indicators of spatial association (LISA) [30] were then visualized using ArcGIS 10.5 software (Figure 2). This analysis revealed that all soil properties were also characterized by strong spatial autocorrelation, whereas the spatial patterns of soil variables were significantly different. High pH values were concentrated in the northwest fields of the village, whereas low values were observed in the east fields of the village. The spatial patterns of soil TN and OM were highly consistent within the study area. The high TN and OM concentrations were located in the southwest of the village, whereas fields east of the village were characterized by low TN and OM concentrations. High AP values were mainly located in the northwest of the village, and few occurred in the southwest fields of the village. The low AP values were concentrated on the eastern side of the village. A high concentration of AK occurred in the central and southern parts of the study area, whereas AK concentrations were low in the northwest and southeast areas of the village. The spatial pattern of the ECa was complex. High values for ECa occurred in the northwest, southwest, and northeast areas of the village, whereas low values were mainly located in the center of the village and did not appear aggregated. Such spatial structure information of soil properties forms the basis of MULTISPATI-PCA.

### 2.3. Creation of the Principal Components

**Figure 3:**
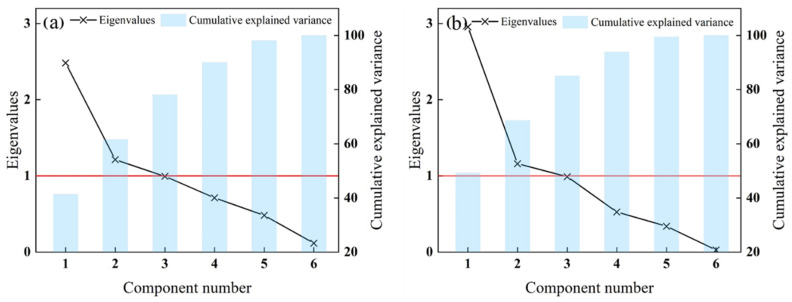
The eigenvalue and cumulative explained variance plots of principal component analysis (PCA) and multivariate spatial analysis based on Moran’s index PCA (MULTISPATI-PCA). (**a**): PCA; (**b**): MULTISPATI-PCA. The red line represents the eigenvalue equal to 1.

The eigenvalue plot (Figure 3a) indicates that the first three PCs explained 78.14% of the total variance. The first three PCs were used for MZ delineation. The eigenvalues and cumulative variance percentages of the SPCs are shown in Figure 3b. The first three SPCs, which explained 85.11% of the total variance, were selected. The results indicated that the PCA and MULTISPATI-PCA approaches performed well in data dimensionality reduction, and three PCs were retained. However, the cumulative variance of the SPCs obtained from MULTISPATI-PCA was higher than that of PCA, which indicated that MULTISPATI-PCA lost less important information from the original data. When comparing PCA and MULTISPATI-PCA in terms of variance and Moran’s I (Table 3), it was apparent that SPCs resulted in lower variance and higher Moran’s I than the PCs, with a variance and Moran’s I of 0.29–1.98 and 0.46–0.68, respectively. These results indicated that MULTISPATI-PCA technology performed better than PCA in variable reduction.

**Figure 4:**
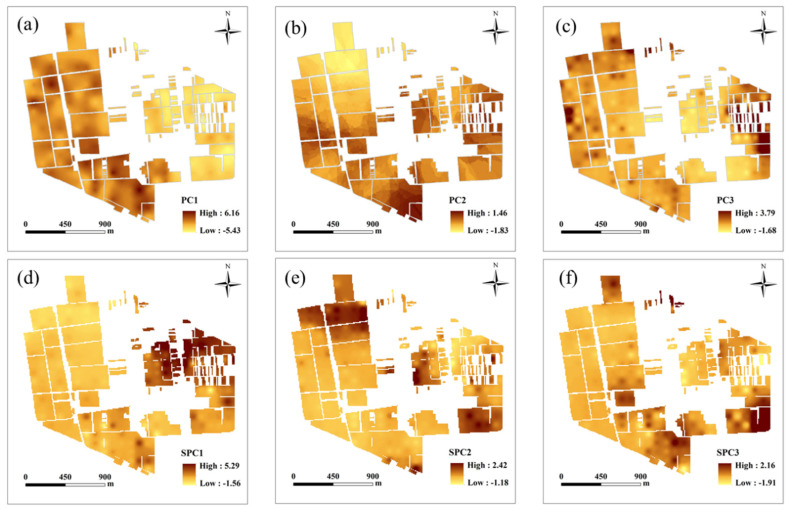
Spatial distribution maps of principal components (PCs) and spatial principal components (SPCs). (**a**): PC1; (**b**): PC1; (**c**): PC3; (**d**): SPC1; (**e**): SPC2; (**f**): SPC3.

In this study, the loadings of PCs and SPCs acted as weights for the soil variables in that component (Table 3). As shown in Table 3, PC1 was dominated by soil TN, OM, and AK, and PC2 was strongly correlated with soil pH. PC3 was positively correlated with ECa. For MULTISPATI-PCA, SPC1 was dominated by soil TN and OM, with loadings of 0.90 and 0.88, respectively, and explained 49.27% of the total variability. SPC2 was strongly correlated with soil pH and AP, with loadings of 0.73 and 0.60, respectively, and explained 19.34% of the total variability. In addition, SPC3 was dominated by soil AK, which accounted for 16.5% of the total variability. The spatial distribution of PCs and SPCs was significantly different (Figure 4) because the two technologies assigned different weights to the soil variables.

### 2.4. Management Zone Delineation and Clustering Models Assessment 

The synthetic variables obtained from the PCA and MULTISPATIAL-PCA approaches were used as inputs, and three clustering algorithms were used to delineate the MZs. ANOVAs (Tukey’s test), VR, and ASC indices were used to evaluate the generated classes. The evaluation results are presented in Table 4. The results reveal that the VR and ASC indices decreased with an increase in the number of clusters. The village is divided into two clusters. The management zone located in the east of the village had high soil fertility, while the soil fertility of MZ in the west of the village was lower. In addition, the yields were significantly different between the two MZs. ANOVAs for soil variables revealed further evidence of the rationality of dividing the village into two classes. The results of the Tukey test revealed that the soil variables were significantly different between the two MZs (Table 5), which further supported the evidence that the optimal number of classes was two.

**Figure 5:**
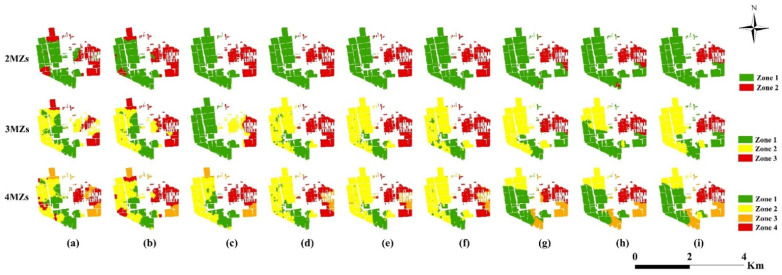
Maps of the MZs defined with the application of nine clustering algorithms. (**a**) All-FCM; (**b**) All-ISODATA; (**c**) All-GMM; (**d**) PCA-FCM; (**e**) PCA-ISODATA; (**f**) PCA-GMM; (**g**) MPCA-FCM; (**h**) MPCA-ISODATA; (**i**) MPCA-GMM. All clustering models’ description are shown in Table 6.

Comparison of the MZs map from PCA (Figure 5c–e) and MULTISPATIAL-PCA technology revealed that the MULTISPATIAL-PCA (Figure 5f–i) provided MZs was more continuous over space, which suggested greater viability for field operations. In addition, the PCA approach and original data for delineating MZs obtained similar ASC and VR values, whereas the use of MULTISPATI-PCA improved the ASC and VR values. These results indicated that the MULTISPATIAL-PCA methods performed the best at MZ delineation. Moreover, compared with the FCM and ISODATA algorithms, the GMM method had the highest ASC and VR values, with the VR values in the range 16.24–20.58%, and the ASC values in the range 0.51–0.57 (Table 4). The results revealed that the GMM algorithm optimized the results of MZ delineation. In addition, when clustering algorithms were used independently to delineate MZs, the values of VR and ASC were 11.32–18.60% and 0.39–0.54, respectively. The PCA and MULTISPATI-PCA technologies, in conjunction with clustering algorithms (model composites), provided better performance for MZ delineation, with VR values in the range 13.35–23.13% and 0.48–0.57, respectively. Moreover, the combination of MULTISPATIAL-PCA with FCM (MPCA-FCM) and GMM (MPCA-GMM) provided the best results, with high VR and ASC values.

**Figure 6:**
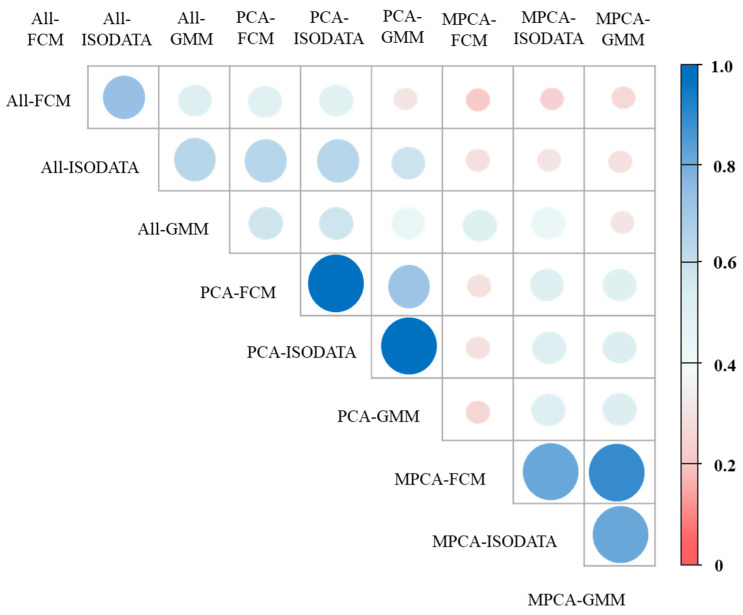
Kappa degrees of agreement between maps obtained by nine clustering models.

The MZ maps produced by the diverse clustering models reveal a stable pattern; however, upon deeper inspection, differences in MZ maps could be found (Figure 5). In addition, the difference between the maps increased by increasing in the number of MZs from two to four. The visual evaluation of the resulting maps was complemented and confirmed by quantitatively evaluating the agreement between the MZ maps using the kappa coefficient (Figure 6). The results showed that the agreement among the MZ maps generated by the different clustering models varied from 0.22–0.94. When using the same variable reduction method, the agreement between the GMM map and two maps of the remaining clustering models (FCM and ISODATA) varied from 0.54–0.94. In addition, the MZ maps obtained by FCM were most similar to the ISODATA maps. When using the same clustering algorithm, the agreement between the map obtained using the raw soil data and the two maps of the remaining variable reduction methods (PCA and MPCA) varied from 0.22–0.60, that is, from weak to moderate agreement. Consequently, the variable reduction method greatly influences the delineation of MZs.

## 3. Discussion

### 3.1. Influencing Factors of Soil Variability

Soil variables are characterized by high spatial variability because of the combined influence of internal factors (parent material and soil texture) as well as external factors (climatic conditions and management practices) [31]. For village farm systems, the different management practices of farmers greatly influence the spatial patterns of soil variables. All soil variables, except pH, showed moderate or strong spatial heterogeneity in this study. Soil pH showed weak variation, with a CV of 3.5%. Similar findings have been reported for the CV of soil pH, ranging from 3.4–14.5% [32,33,34]. The concentrations of OM and TN in the village were higher than those reported by [35], which may be related to the return of straw to the field. Many previous studies have confirmed that straw return effectively improves soil OM levels [36,37]. Soil AP and AK were characterized by high spatial variability in this study, with CVs of 91.43% and 22.37%, respectively, which is consistent with the findings of [38]. This phenomenon can be explained in two ways. First, fertilization practices significantly influence soil available nutrients (e.g., AP and AK), which contributes to the accumulation of soil available nutrients [39]. The fertilizer application rate varied greatly in intensive farming systems, resulting in high variability of soil AP and AK [11]. In addition, the crop rotation systems in this study are complicated; winter wheat and summer maize are the main cropping systems, and there are a few fields where wheat is rotated with peanuts or soybeans. This may also be one reason for the high variability of soil nutrient concentrations [40].

### 3.2. The Spatial Dependence of Soil Properties

Understanding the spatial variability and spatial patterns of soil variables is the basis for accurately delineating MZs. All soil variables had moderate to strong spatial dependence. The wheat yield had a relatively similar spatial dependence ranking to the soil variables, suggesting that these soil properties may be strong factors in determining yields. This is similar to the findings of Yuan et al., who reported that soil OM, AP, AK, and EC had a significant influence on wheat yield [41]. In addition, large nugget values of soil AK and yield may suggest that the soil sampling interval was too large to capture the spatial autocorrelation and dependence of soil variables well [42]. Therefore, a smaller sampling interval is recommended to characterize the spatial pattern of soil AK and yield more accurately. Kerry and Oliver reported that the soil sampling interval should be less than half the range of the average variogram [29]. Nonetheless, the selected soil sampling distance in this study was appropriate for accurately capturing the spatial variability characteristics of the measured soil variables. For soil pH, TN, OM, AP and ECa, an appropriate reduction of sampling points may be possible to still characterize the spatial dependence and reduce costs, which requires further investigation.

The local Moran’s I measured the spatial correlation between objects and their neighbors, and analyzed the local feature differences of spatial objects, reflecting the spatial heterogeneity in local areas. The soil properties were characterized by clear spatial clustering, which is consistent with the findings of Fu et al. [43]. The spatial patterns of soil TN and OM were highly consistent, resulting in a significant positive correlation between soil TN and OM [44]. A previous study reported that soil type had a significant impact on nutrient leaching, possibly because the movement of these nutrients through water is influenced by soil properties that determine their retention and transport [45]. Two soil types, sand, and loam, occurred in the village, which partially explains the different spatial patterns of soil TN and OM between the east and west sections of the village. Loam is mainly distributed in the west of the village, where high–high value clustering for soil OM and TN accumulated. Sandy soil largely characterized the eastern section of the study area, where low–low clustering for soil OM and TN occurred. In addition, the fields where crops are planted intensively have high fertility, while the fields in the east of the village are planted with a large number of vegetable greenhouses, which may cause soil acidification, salinization, and crop labor and lack of nutrients [46]. Therefore, the soil fertility in the eastern part of the village is low. Ren et al. reported similar conclusions that the OM content was closely related to the soil type, and the OM concentration in loam soil was higher than that in sandy soil [47]. In this study, the high AP concentration was concentrated in the east of the village, whereas AP concentrations in the northwest and southwest of the study area were low. Therefore, proper P supplementation is likely to increase production significantly. Likewise, attention and improved K management of the different agricultural practices should be considered in the northeast, southeast, and northwest sections of the study area, where a low AK content occurred (Figure 2).

### 3.3. The Evaluation of Clustering Model Composites

The ASC is a commonly used index for clustering evaluation in machine learning. However, a previous study reported that the ASC values were high even when the algorithms could not form different clusters. This indicates that it is not adequate to evaluate clustering results using the ASC index exclusively [48]. This finding was also reflected in the present study. ANOVA, VR, and ASC metrics were used in this study to evaluate the MZ delineation performance of clustering models. The results indicate that the various metrics were not equivalent but were complementary. When comparing PCA and MULTISPATI-PCA from the perspective of variance and spatial dependence, SPC1 obtained a lower variance and higher Moran’s I than PC1. The results showed that the MULTISPATI-PCA approach increased the degree of spatial autocorrelation because the technology incorporated spatial constraints that considered the spatial dependence of the measured variables. In addition, the soil MZs produced by the combination of MULTISPATI-PCA and three clustering algorithms was more continuous over space, which was more conducive to the implementation of on-the-ground agricultural practices. Several previous studies have reported similar results [16,17,49]. Therefore, the MULTISPATI-PCA was more conducive to selecting PCs needed for MZ delineation than the traditional PCA. Similar findings were reported by Córdoba et al. [50], who applied PCA and MULTISPATI-PCA in conjunction with FCM to delineate MZs in Argentina. In addition, the spatial distribution maps of PCs and SPCs differed, owing to the different weights for the soil variables. PC1 was dominated by soil TN, OM, AP, and AK, whereas SPC1 was dominated by soil TN and OM, which led to a significant difference in the spatial maps. Similarly, the spatial maps of PC2 and SPC2, PC3, and SPC3 were quite different. These results are similar to those of Ohana-Levi et al. [18]. They reported that the relative weight assigned to each variable greatly influenced the results of the clustering model and that the final shape of the MZs was determined by the spatial pattern of variables with a higher weight. Consequently, in this study, there was a weak agreement between the MZs map of PCA and MULTISPATI-PCA (Figure 6).

Comparing the MZ maps of FCM, ISODATA, and GMM, the results demonstrate that the map of FCM was similar to that of the ISODATA approach, which might be because these two clustering algorithms delineated MZs based on similarity or dissimilarity distance. Thus, the two methods are significantly influenced by the sample distance. Several previous studies have reported similar conclusions [11,21,48]. However, the GMM map was different from that of FCM and ISODATA. As a model-based clustering algorithm, the data distribution significantly influences the MZs map. The GMM algorithm is not sensitive to the sample distance and is suitable for sample data of various shapes. In addition, Haghverdi et al. reported that GMM might be more suitable for clustering than FCM if the clusters have different sizes [22]. Moreover, this study demonstrated a high MZ delineation potential of the GMM algorithm for intensive farming systems as indicated by the highest VR and ASC values.

Gavioli et al. applied SC, PCA, and MULTISPATI-PCA with the FCM algorithm to delineate MZs, and they confirmed that the combination of MPCA-SC and FCM provided the best performance of MZ delineation [16]. In addition, Ohana-Levi et al. compared the MZ delineation effectiveness of six clustering model composites with the results indicating that the model composites considering spatial variability produced better results [18]. Similar results were obtained in this study, and both MPCA-GMM and MPCA-FCM effectively improved the performance of MZ delineation. There were different degrees of spatial variability for the soil variables in this study, which likely influenced the results of the model composites. Therefore, the spatial dependence was quantified in deter each soil variable weight before delineating the MZs. Deeper exploration revealed that the clustering algorithm performance might be different among study cases with differing ecological factors. This might be because data sets from different study areas have unique characteristics, and it may be difficult to find a clustering method that works best in some cases. Therefore, comparative studies are needed to further assess the performance of different clustering model composites in delineating MZs in different study cases.

## 4. Materials and Methods

### 4.1. Site Description

**Figure 7:**
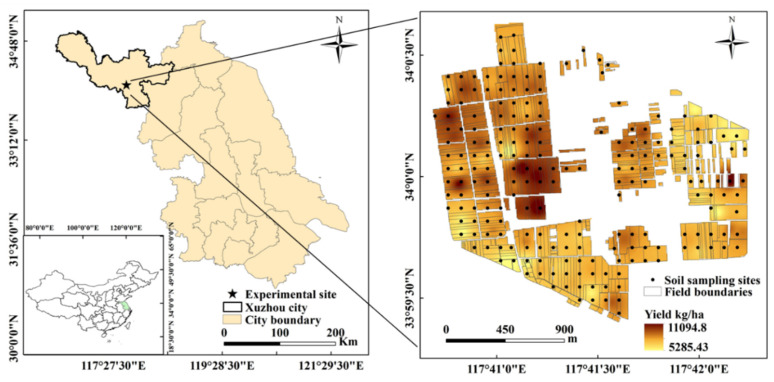
Location of the study area and the sampling grids used to guide soil sample collection of the experimental site (Zhaoji village, Jiangsu, China).

The village selected for this study was Zhaoji Village in Suining County, Jiangsu Province, China (33°59′38″ N, 117°40′46″ E). The area’s annual temperature and precipitation are 14 °C and 922 mm, respectively, and it has a warm monsoon temperate terrestrial climate. Summers are typically characterized by significant rainfall, often accounting for as much as 56% of the annual precipitation. The other seasons have an average precipitation of 135 mm. Supplemental irrigation is frequently required. The soil in the village was sand and loam. The cropping systems in this study area are mainly winter wheat and summer maize, with a few farmers planting soybeans and peanuts. All straw was returned to the field after wheat harvest. The cropped area of this study comprised of approximately 209 ha distributed across 458 fields. The average area of each field was 0.45 ha (Figure 7).

### 4.2. Data Collection and Measurements

#### 4.2.1. Soil Chemical Properties

Soil sampling was carried out after the wheat harvest in 2020. A total of 208 soil samples were collected to a 0.3 m depth with a grid of 100 m × 100 m designed and delineated in ArcGIS 10.5 (ESRI, Redlands, CA, USA). The project area included the entire village. The spatial distribution of the soil samples is shown in Figure 7. The 0–0.3 m depth was chosen because it is the soil layer most influenced by crop nutrient fixation and nutrient management practices. Five sub-samples were mixed into a composite sample within a 2 m radius around the location of the sample point and used as representative soil samples for each site. The soil samples were air-dried and ground to pass through a 1 mm sieve. Soil variables were analyzed using national standard analysis methods [51]. 

#### 4.2.2. Apparent Soil Electrical Conductivity Data

Soil ECa (ms m^−1^) was measured after wheat harvest using an EM38-MK2 ground conductivity meter (Geonics Ltd.; Ontario, Canada). The instrument consists of a transmitting coil and two receiver coils and it can be used in either horizontal or vertical modes [52]. In this study, the EM38-MK2 was used in the vertical dipole mode, thus providing readings at two depths, from 0–0.75 m and from 0–1.5 m for the 0.5 m and the 1.0 m transmitter coils, respectively. The sensor was calibrated at the height of 1.5 m above the ground in both the horizontal and vertical dipole directions before taking field measurements. After the calibration procedures were completed, the sensor was held perpendicular to the ground by hand to provide vertical dipole readings. The ECa of each soil sample was the average value of the readings within a circle of 2 m radius. Estimated ECa at un-sampled locations was carried out using the spatial interpolation methods with the ArcGIS software.

#### 4.2.3. Yield Data

Wheat yield data for each sample point were obtained by hand-harvesting 1 m^2^ sample areas. The ordinary kriging (OK) interpolation method was used to develop a yield map across the village and to estimate the yield data for un-sampled points. 

### 4.3. Clustering of Variables

#### 4.3.1. Statistical and Geostatistical Analysis

**Figure 8:**
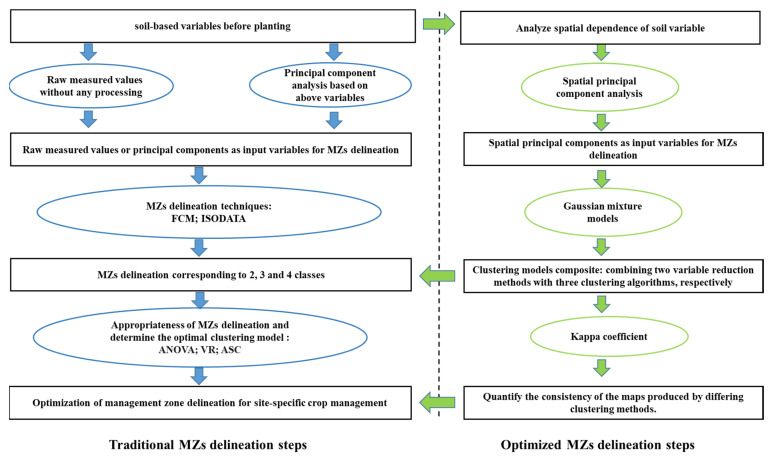
Flow chart of the optimized MZ delineation in this work.

The classical non-spatial statistics of the measured values were calculated using SPSS 25 (IBM SPSS Statistics, Armonk, NY, USA). The local autocorrelation index of Moran was calculated using ArcGIS software (ESRI, Redlands, CA, USA) to exclude inliers and characterize the spatial dependence of soil variables. Because a highly skewed dataset negatively affects the spatial structure estimates [29], a logarithmic transformation was used to improve the normality of the data. Semivariograms were computed using the GS+ software in order to characterize the spatial autocorrelation of soil variables and provide basic parameters required for the OK approach. The resulting interpolation, estimation of values at un-sampled locations and development of the map, was performed using ArcGIS software, Geostatistical Analysis Extension. The resulting spatial maps of soil properties were used to delineate MZs, and the yield map was used to determine the optimal classes and evaluate the results of different clustering models. The detailed process is illustrated in Figure 8.

#### 4.3.2. Variable Reduction

This study compared two approaches for selecting variables and raw, measured values were used to define MZs. The PCA and MULTISPATI-PCA approaches produced new uncorrelated variables from the raw dataset and thus helped achieve data dimensionality reduction. In addition, the resulting MZ maps from the raw data were then used to explore the influence of variable reduction and weight assignment by the two different approaches to MZ delineation.

The PCA approach transforms the original variables into a set of uncorrelated variables called principal components (PCs) using orthogonal transformations [53]. Raw values for the six soil variables were standardized by z-score standardization using the SPSS software and used as input for PCA. The PCs were then calculated based on the standardized soil variables. The number of PCs selected were based on the principle that the cumulative variance is greater than 70% [16]. Moreover, the eigenvalues of each PC represent their contribution to explaining the total variability. The eigenvalues of the PCs were required to be greater than 1. If both criteria were met, the PCs were retained and used to delineate the MZs.

The MULTISPATI-PCA approach introduced a spatial weight matrix to traditional PCA, which thus considered the spatial dependence of soil-measured values. The number of selected spatial principal components (SPCs) were based on the principle that the cumulative variance is greater than 70%. Moreover, the eigenvalues of the SPCs were required to be greater than 1. The SPCs obtained from the MULTISPATI-PCA approach were then used as inputs to identify the homogeneous zones.

PCA and MULTISPATI-PCA approaches were performed using SPSS and GeoDa1.14 [30]. The GeoDa software provides a user-friendly and graphical interface that facilitates exploratory analysis of spatial data. This software was also used to generate a spatial weight matrix and to perform spatial dependency analysis of soil variables by calculating Moran’s index.

#### 4.3.3. Clustering Algorithm for Delineating MZs

The study selected two clustering methods based on similarity distances and one model-based clustering method to delineate the MZs. These approaches are FCM, ISODATA, and GMM.

The FCM approach is an unsupervised clustering algorithm and the preferred technology for MZ delineation [21]. In addition, this method obtains the membership degree of each object to all class centers by optimizing the objective function, thereby determining the class of the object to achieve the purpose of automatically classifying sample data. The village was delineated into two to four clusters using MZA software (Agriculture Research Service, University of Missouri-Columbia, Missouri, USA) to accommodate the diverse systems of agricultural production. In addition, the maximum iteration, stopping criterion, and fuzziness exponent were set to 300, 0.0001, and 1.5, respectively.

The ISODATA algorithm includes two operations based on the K-means algorithm by merging and splitting the clustering results. The approach begins with randomly assigning data samples to different groups. The mean for each group was then calculated, and the remaining objects were iteratively grouped by minimizing the distance from each objective to the group mean. In addition, the means were recalculated for each iteration, and the objectives were reclassified according to the new group mean. Importantly, the ISODATA approach can merge and split clusters according to the similarity between the groups and the specified minimum group size until it reaches the maximum iteration number [54]. In this study, the ISODATA approach was performed using the Iso Cluster unsupervised classification function of ArcGIS 10.5, with a minimum class size of 20, and a sampling interval of 10.

Generally, a mixture model can use any probability distribution [55]. The Gaussian mixture model (GMM) was selected for this study because it has good mathematical properties and calculation performance. The GMM is a parametric probability density model that assumes that all data samples are generated from the mixture parameters of a Gaussian distribution. A mixture model can be considered as an extension of the K-means clustering algorithm, which incorporates the covariance structure of the data. In addition, the GMM approach uses a Gaussian distribution as a parametric model and expectation-maximization algorithm for training [27].

The data set used in this study comprised soil observations, and the parametrized component density was a multidimensional Gaussian density. The calculation steps of the multivariate Gaussian distribution were previously described by Hu et al. [26]. The Gaussian mixture density can be expressed by Equation (1):

(1) $$p_{k|n}\;=\;\frac{\pi_{k}\frac{1}{\prod_{i=1}^{I}\sqrt{2\pi }\sigma_{i}^{\left(k\right)}}exp\left(-\sum_{i}^{I}{\left(\mu_{i}^{\left(K\right)}-x_{i}^{\left(n\right)}\right)}^{2}/2{\left(\sigma_{i}^{\left(k\right)}\right)}^{2}\right)}{\sum_{k^{\prime }}\pi_{k^{\prime }}\frac{1}{\prod_{i=1}^{I}\sqrt{2\pi }\sigma_{i}^{\left(k^{\prime }\right)}}exp\left(-\sum_{i}^{I}{\left(\mu_{i}^{\left(k^{\prime }\right)}-x_{i}^{\left(n\right)}\right)}^{2}/2{\left(\sigma_{i}^{\left(k^{\prime }\right)}\right)}^{2}\right)}$$

where *p* represents mixture density, *k* represents the number of Gaussian distributions, *i* and *n* represent the serial number of the data dimension and data sequences, respectively, *I* represents the total number of data dimensions, $\pi_{k}$ is the weight, and the $\mu_{i}^{\left(K\right)}$ and $\sigma_{i}^{\left(k\right)}$ terms are the mean and variance of the Gaussian distribution, respectively. Finally, $x_{i}^{\left(n\right)}$ represents the data points.

For the GMM algorithm, the mean and variance are important and are estimated using an expectation-maximization algorithm. The iterative formula for the mean $\mu_{i}^{\left(K\right)}$ and variance $\sigma_{i}^{\left(k\right)}$ are presented in Equations (2) and (3), respectively. The weights $\pi_{k}$ were calculated using Equation (4):

(2) $$\mu_{k}^{’}\;=\;\frac{\sum_{n}p_{k|n}x_{n}}{\sum_{n}p_{k|n}}$$

(3) $$\sigma_{i}^{2\left(k\right)}\;=\;\frac{\sum_{n}p_{k|n}{\left(x^{\left(n\right)}-\mu_{i}^{\left(k\right)}\right)}^{2}}{\sum_{n}p_{k|n}}$$

(4) $$\pi_{k}\;=\;\frac{\sum_{n}p_{k|n}}{\sum_{k}\sum_{n}p_{k|n}}$$

The GMM approach divides the dataset into different groups as the iterations proceed. Scikit-learn [56], a Python machine-learning library, was used to perform the GMM approach for MZ delineation. In this study, the results of the variable reduction were used as inputs to the different clustering algorithms for delineating MZs. Specific descriptions of these nine clustering model composites (two variable reduction methods combined with three clustering algorithms) are shown in Table 6.

**Table 6:** Details on the nine clustering models that were used in the analysis.

Model Composite	Model Description
**All-FCM:** All measured values with FCM algorithm	Raw values for the six soil variables were used as input, and the FCM algorithm was applied on the raw values.
**All-ISODATA:** All measured values with fuzzy ISODATA algorithm	Raw values for the six soil variables were used as input, and the ISODATA algorithm was applied on the raw values.
**ALL-GMM:** All measured values with GMM algorithm	Raw values for the six soil variables were used as input, and the GMM algorithm was applied on the raw values.
**PCA-FCM:** Principal components by PCA with FCM algorithm	Raw values for the six soil variables were standardized and used as input of PCA, and the FCM algorithm was applied on the principal components
**PCA-ISODATA:** Principal components by PCA with ISODATA algorithm	Raw values for the six soil variables were standardized and used as input of PCA, and the ISODATA algorithm was applied on the principal components.
**PCA-GMM:** Principal components by PCA with GMM algorithm	Raw values for the six soil variables were standardized and used as input of PCA, and the GMM algorithm was applied on the principal components.
**MPCA-FCM:** Spatial principal components by MULTISPATI-PCA with FCM algorithm	Raw values for the six soil variables were standardized and used as input of MULTISPATI-PCA, and the FCM algorithm was applied on the spatial principal components.
**MPCA-ISODATA:** Spatial principal components by MULTISPATI-PCA with ISODATA algorithm	Raw values for the six soil variables were standardized and used as input of MULTISPATI-PCA, and the ISODATA algorithm was applied on the spatial principal components.
**MPCA-GMM:** Spatial principal components by MULTISPATI-PCA with GMM algorithm	Raw values for the six soil variables were standardized and used as input of MULTISPATI-PCA, and the GMM algorithm was applied on the spatial principal components.

#### 4.3.4. Assessment of the Clustering Model

In this study, analysis of variance, variance reduction (VR), and average silhouette coefficient (ASC) were selected to determine the optimal class number and evaluated the MZ delineation performance of different clustering models. In addition, the kappa coefficient was used to assess the degree of consistency among the MZ maps.

Tukey’s range test was performed to determine whether the obtained groups showed significant differences in yield and soil variables in this study.

The VR index was calculated assuming that the sum of the yield variances of different MZs would be smaller than the variance of the total study area (Equation (5)). In addition, the VR index reflected the degree of homogeneity within the MZ, to a certain extent.

(5) $$\mathrm{VR}\;=\;\left(1-\frac{\sum_{i=1}^{c}W_{i}\times V_{mz_{i}}}{V_{field}}\right)\;\times \;100$$

where *c* represents the number of MZs; $W_{i}$ represents the ratio of the area of the *i*-th MZ to the total area of the study area; $V_{mz_{i}}$ and $V_{field}$ are the yield variance of the *i*-th MZ and the whole study area, respectively.

The ASC coefficient is an evaluation index that reflects the degree of similarity within the MZ and the separation between MZs. The silhouette coefficient was calculated using Equation (6).

(6) $$S\left(i\right)\;=\;\frac{b\left(i\right)-a\left(i\right)}{max\left(a\left(i\right),b\left(i\right)\right)}$$

where *i* is a data sample in a class, a(i) represents the average distance of sample *i* to the sample in the same class, which represents the degree of similarity within the MZ, b(i) represents the average distance of *i* to the points in other clusters, which shows the degree of separation between MZs. S(i) is the average value of the ASC [57]. In addition, the ASC ranged from −1–1. A value close to 1 indicates that the clustering model achieved a good performance of MZ delineation, whereas the values approaching −1 represent an incorrect grouping.

The kappa coefficient was used to evaluate the agreement between the cluster results produced by the nine clustering methods. The kappa coefficient was calculated based on the confusion matrix, and the calculation formula given in Equation (7).

(7) $$K_{a}\;=\;\frac{p_{0}-p_{e}}{1-p_{e}}$$

where $K_{a}$ is the kappa value, $p_{0}$ is the ratio of the sum of diagonal elements to the sum of the total elements, $p_{e}$ is calculated by Equation (8):

(8) $$p_{e}\;=\;\frac{\sum_{k=1}^{N}\left(\sum_{j=1}^{N}a_{kj}\times \sum_{i=1}^{N}a_{ik}\right)}{{\left(\sum_{i=1}^{N}\sum_{j=1}^{N}a_{ij}\right)}^{2}}$$

where *i* and *j* represent the row and column of the confusion matrix, respectively, *N* is the order of the confusion matrix, $a_{ij}$ represents the value of the *i*-th row and *j*-th column. The degree of agreement ($K_{a}$) was divided into five categories [58]: $0\;<\;K_{a}\;\leq \;0.2$ indicates that the clustering results have no agreement; $0.2\;<\;K_{a}\;\leq \;0.4$ indicates that the degree of agreement is weak among MZ maps; $0.4\;<\;K_{a}\;\leq \;0.6$ indicates the degree of consistency is moderate; $0.6\;<\;K_{a}\;\leq \;0.8$ indicates that the degree of consistency is strong; and $0.8\;<\;K_{a}\;\leq \;1$ indicates that clustering results are highly consistent.

## 5. Conclusions

In conclusion, the clustering model composites greatly improved the efficiency of delineating MZs. PCA and MULTISPATI-PCA greatly influenced the clustering maps, because of the different weights assigned to the soil variables. The final MZs map strongly reflected the spatial pattern of soil variables with high weights. In addition, the MULTISPATI-PCA method improved the performance of MZ delineation. The maps produced by considering the spatial structure information of the soil variables were more uniform and spatially continuous, which is expected to facilitate practical field operations. The study demonstrates that the GMM algorithm has great MZ delineation potential for intensive farming systems. A variety of multivariate clustering model composites were applied to delineate MZs for site-specific fertilization practices. This demonstrated that the model composites provided a better performance of MZ delineation than by using clustering algorithms independently. Moreover, MPCA-GMM and MPCA-FCM produced the best results in this study, with the highest VR and ASC values. Meanwhile, the soil variables and yield data were significantly different between the MZs generated by the two clustering models. The Kappa coefficient of the different clustering algorithms varied from 0.54–0.94 (moderate to strong), whereas the Kappa coefficient of the different variable reduction approaches varied from 0.22–0.60 (weak to moderate). The results indicated that, compared with the clustering algorithm, the influence of the variable weight assignment on MZ delineation could not be ignored. In addition, the model composite for clustering should be flexible and possibly composed of different algorithms, enabling the selection of an optimal approach for MZ delineation. Model performance may differ among study cases with different intrinsic factors. Therefore, comparative research is needed to further assess the performance of these clustering models in delineating MZs at different experimental sites with varying soil and weather conditions.

## Figures and Tables

**Table 1:** Summary statistics of the studied variables.

Variables	Min	Max	Mean	SD	CV/%	Skewness	Kurtosis	Moran’s I
pH	7.51	8.46	8.10	0.28	3.50	−0.46	3.73	0.38 *
TN (g kg^−1^)	0.59	1.88	1.23	0.24	19.51	−0.11	2.64	0.42 *
OM (g kg^−1^)	6.87	28.01	17.27	3.62	20.96	−0.27	2.92	0.53 *
AP (mg kg^−1^)	5.85	261.77	31.16	28.49	91.43	1.41	5.93	0.39 *
AK (mg kg^−1^)	89.00	323.15	207.30	46.38	22.37	0.12	2.58	0.24 *
ECa (ms m^−1^)	10.15	51.03	18.32	6.05	33.33	1.11	4.80	0.35 *
Yield (t ha^−1^)	5.25	10.95	8.33	1.27	16.16	0.07	2.63	0.11 *

TN: total nitrogen; OM: organic matter; AP: available phosphorus; AK: available potassium; ECa: soil apparent electrical conductivity; SD: standard deviation; CV: coefficient of variation; Moran’s I: Global Moran’s Index; * represent the value is significant at 0.01 level.

**Table 2:** Semivariogram models and related geostatistical parameters for characterizing the spatial dependence of soil variables.

Variables	Model	Nugget (C0)	Sill (C + C0)	SDC	Range (m)	R^2^
pH	Gaussian	0.01	0.03	Moderate	866.03	0.94
TN	Gaussian	0.00	0.04	strong	214.77	0.80
OM	Gaussian	0.01	6.65	Strong	180.13	0.86
AP	Exponential	0.18	0.68	Moderate to strong	1227.43	0.85
AK	Exponential	1029	2417	Moderate	1770.00	0.94
ECa	Spherical	0.02	0.05	Moderate	849.64	0.92
yield	Exponential	1000	168,4000	Strong	945.00	0.84

TN: total nitrogen; OM: organic matter; AP: available phosphorus; AK: available potassium; ECa: soil apparent electrical conductivity; SDC: spatial dependency class, ranking of spatial dependence The SDC (Nugget/Sill × 100%) of <25%, 25–75%, and >75% reveals strong, moderate, and weak spatial dependence, respectively [4].

**Table 3:** Loadings for the variables in the principal components (PCs) and spatial principal components (SPCs) and statistics of the PCs and SPCs.

Component	pH	TN	OM	AP	AK	ECa	Variance	Moran’s I
**PCA**								
PC1	0.33	0.94	0.95	−0.73	0.70	0.18	6.23	0.62 *
PC2	−0.85	0.16	0.15	0.45	0.40	0.18	1.77	0.24 *
PC3	0.12	0.02	−0.06	0.03	−0.21	0.96	0.94	0.33 *
**MULTISPATI−PCA**								
SPC1	−0.34	0.90	0.88	0.58	0.02	0.67	1.98	0.68 *
SPC2	0.73	−0.22	−0.20	0.60	0.27	0.40	0.47	0.52 *
SPC3	−0.11	0.10	0.03	−0.21	0.96	−0.06	0.29	0.46 *

TN: total nitrogen; OM: organic matter; AP: available phosphorus; AK: available potassium; ECa: soil apparent electrical conductivity; PC: principal component; SPC: spatial principal component; * represent the value is significant at 0.01 level.

**Table 4:** Results of the evaluation of the clustering models in the generation of two, three, and four classes in terms of the ANOVA (Tukey’s test), variance reduction (VR) index, and average silhouette coefficient (ASC).

	C1	C2	VR (%)	ASC	C1	C2	C3	VR (%)	ASC	C1	C2	C3	C4	VR (%)	ASC
All-FCM	a	b	13.64	0.47	a	b	c	12.14	0.42	a	b	c	ac	10.96	0.38
All- ISODATA	a	b	11.32	0.39	a	b	c	10.67	0.37	a	b	b	c	13.47	0.28
All-GMM	a	b	18.60	0.54	a	b	c	17.68	0.48	a	b	c	d	16.05	0.36
PCA-FCM	a	b	15.36	0.48	a	b	b	11.61	0.37	a	b	c	bc	8.47	0.31
PCA- ISODATA	a	b	13.35	0.49	a	a	b	10.67	0.32	a	b	c	bc	9.56	0.26
PCA-GMM	a	b	16.24	0.51	a	b	c	12.11	0.45	a	b	b	c	10.34	0.35
MPCA-FCM	a	b	**23.13**	0.56	a	b	c	18.71	0.51	a	b	a	c	**18.69**	**0.44**
MPCA- ISODATA	a	b	19.08	0.52	a	b	b	17.07	0.44	a	b	c	d	15.94	0.30
MPCA-GMM	a	b	20.58	**0.57**	a	b	c	**20.32**	**0.56**	a	b	c	b	17.70	**0.44**

C1, C2, C3, and C4 represent the classes generated by each clustering method. Values marked in bold and underlined highlight the best model for delineating MZs, while non-bold, underlined marks define the second-best model for delineating management zones.

**Table 5:** Tukey’s test for the soil variables when the village was divided into two classes, considering classes defined with the nine clustering models.

Model	pH	TN	OM	AP	AK	ECa
ALL-FCM	**	**	**	**	**	*
ALL-ISODATA	**	**	**	**	**	**
ALL-GMM	**	**	**	*	**	**
PCA-FCM	**	**	**	**	**	**
PCA-ISODATA	**	**	**	**	**	**
PCA-GMM	**	**	**	**	**	**
MPCA-FCM	**	**	**	**	**	**
MPCA-ISODATA	**	**	**	**	**	**
MPCA-GMM	**	**	**	**	**	**

TN: total nitrogen; OM: organic matter; AP: available phosphorus; AK: available potassium; ECa: soil apparent electrical conductivity; **: significant difference between the averages of all classes at the 0.01 level; *: significant difference between the averages at the 0.05 level.

## Data Availability

Data available on request due to restrictions eg privacy or ethical.
